# Life cycle and post-mortem ingrowth patterns of a leadless pacemaker system

**DOI:** 10.1093/europace/euae013

**Published:** 2024-01-18

**Authors:** Hermann Blessberger, Daniel Kiblboeck, Claudia Grosse, Petar Noack, Jakob Ebner, Jakob Boetscher, Julian Maier, Stefan Schwarz, Christian Reiter, Alexander Fellner, Michael Lichtenauer, Joerg Kellermair, Juergen Kammler, Karim Saleh, Clemens Steinwender

**Affiliations:** Department of Cardiology, Kepler University Hospital, Krankenhausstraße 9, 4020 Linz, Austria; Medical Faculty of the Johannes Kepler University Linz, Altenberger Straße 64, 4040 Linz, Austria; Department of Cardiology, Kepler University Hospital, Krankenhausstraße 9, 4020 Linz, Austria; Medical Faculty of the Johannes Kepler University Linz, Altenberger Straße 64, 4040 Linz, Austria; Medical Faculty of the Johannes Kepler University Linz, Altenberger Straße 64, 4040 Linz, Austria; Institute of Clinical Pathology and Molecular Pathology, Kepler University Hospital, Linz, Austria; Medical Faculty of the Johannes Kepler University Linz, Altenberger Straße 64, 4040 Linz, Austria; Institute of Clinical Pathology and Molecular Pathology, Kepler University Hospital, Linz, Austria; Department of Cardiology, Kepler University Hospital, Krankenhausstraße 9, 4020 Linz, Austria; Medical Faculty of the Johannes Kepler University Linz, Altenberger Straße 64, 4040 Linz, Austria; Department of Cardiology, Kepler University Hospital, Krankenhausstraße 9, 4020 Linz, Austria; Medical Faculty of the Johannes Kepler University Linz, Altenberger Straße 64, 4040 Linz, Austria; Department of Cardiology, Kepler University Hospital, Krankenhausstraße 9, 4020 Linz, Austria; Medical Faculty of the Johannes Kepler University Linz, Altenberger Straße 64, 4040 Linz, Austria; Department of Cardiology, Kepler University Hospital, Krankenhausstraße 9, 4020 Linz, Austria; Medical Faculty of the Johannes Kepler University Linz, Altenberger Straße 64, 4040 Linz, Austria; Department of Cardiology, Kepler University Hospital, Krankenhausstraße 9, 4020 Linz, Austria; Medical Faculty of the Johannes Kepler University Linz, Altenberger Straße 64, 4040 Linz, Austria; Department of Cardiology, Kepler University Hospital, Krankenhausstraße 9, 4020 Linz, Austria; Medical Faculty of the Johannes Kepler University Linz, Altenberger Straße 64, 4040 Linz, Austria; Department of Internal Medicine II, Paracelsus Medical University, Salzburg, Austria; Department of Cardiology, Kepler University Hospital, Krankenhausstraße 9, 4020 Linz, Austria; Medical Faculty of the Johannes Kepler University Linz, Altenberger Straße 64, 4040 Linz, Austria; Department of Cardiology, Kepler University Hospital, Krankenhausstraße 9, 4020 Linz, Austria; Medical Faculty of the Johannes Kepler University Linz, Altenberger Straße 64, 4040 Linz, Austria; Department of Internal Medicine II, Paracelsus Medical University, Salzburg, Austria; Department of Cardiology, Kepler University Hospital, Krankenhausstraße 9, 4020 Linz, Austria; Medical Faculty of the Johannes Kepler University Linz, Altenberger Straße 64, 4040 Linz, Austria; Department of Cardiology, Kepler University Hospital, Krankenhausstraße 9, 4020 Linz, Austria; Medical Faculty of the Johannes Kepler University Linz, Altenberger Straße 64, 4040 Linz, Austria; Department of Internal Medicine II, Paracelsus Medical University, Salzburg, Austria

**Keywords:** Leadless pacemaker, Causes of death, Time to death, Inflammation, Fibrosis, Encapsulation

**What’s new?**

- About 45% of our patients with a leadless pacemaker system (LPS) died from cardiac causes. In our study there were no signs of pacemaker-induced arrhythmic death.
- Encapsulation of the LPS varied and was not time-dependent.
- The histological analysis of eight post-mortem tissue samples revealed a fibrous capsule with varying degrees of lymphocyte-rich inflammation adjacent to the LPS body without any signs of endothelialization.

Leadless pacemaker systems (LPS) have become an established therapy in selected patients. However, there is still debate about the feasibility and method of device retrieval after battery depletion or in cases of systemic endocarditis where device infection cannot be ruled out, especially years after initial implantation.^1^ To date, data are still limited on how long patients wear their LPS, what causes their death, and the extent to which LPS become overgrown with cardiac tissue over time. Overgrowth may affect the feasibility of retrieval and electrical device performance. In the future, it may become possible to precisely control tissue coverage by applying particular cell- or fibrosis-repellent surface coatings or treatments.^2^

In this retrospective single-centre cohort study, all patients who had undergone a Micra™ LPS (Medtronic Inc., Minneapolis, USA) implantation at our hospital were included. The study was approved by the local ethics committee. Patient survival status was determined by contacting local registration authorities, and the cause of death was obtained from the Austrian National Statistical Institute and by reviewing autopsy reports. The ‘service period’ was defined as the time from LPS implantation to either the last follow-up or the patient’s death. A thorough pathohistological examination including immunohistological stains was performed by two experienced pathologists when post-mortem myocardial tissue blocks containing the LPS were available.

The study cohort comprised 283 patients (LPS implant between December 2013 and July 2020, average age 79.2 years, 36.4% female), of whom 71 (25.1%) died during a median follow-up of 2.7 (IQR: 1.5–4.4, range: 0–7.1) years. The indications for leadless pacing were atrial fibrillation with slow conduction (41.3%), high-grade AV block (33.6%), sick sinus syndrome with a low anticipated ventricular pacing rate (14.5%), and others (10.6%). The median age at death was 85.9 years (IQR: 79.9–89.7, range: 61.8–99.1 years). The median service period was 1.9 years (IQR: 1.1–3.1, range: 0–5.7 years). Death was due to cardiac in 32 (45.1%) and non-cardiac conditions in 39 (54.9%) patients. Cardiac causes comprised: acute coronary syndrome (*n* = 6), coronary heart disease (*n* = 11), heart failure (*n* = 2), ischaemic cardiomyopathy (*n* = 2), aortic stenosis (*n* = 1), and other cardiac diseases (*n* = 10). Non-cardiac conditions included different types of cancer (*n* = 12), renal disease (*n* = 6), neurological conditions (*n* = 5), chronic obstructive pulmonary disease (*n* = 1), infections other than device infection (*n* = 6), as well as others (*n* = 9). No death was classified as device-related or as arrhythmic death that might have been associated with leadless pacing. During follow-up, LPS dysfunction was a rare event (three cases of raised pacing thresholds, all within three months after implant), and two LPS could be successfully retrieved (each about one month after the initial implantation). No LPS infections were noted in our cohort, and no relapses were detected in patients with LPS implantation after previous lead extraction due to device infection. The histological analysis of eight (11.3%) post-mortem tissue samples (service period: 18–2083 days, age 75–87 years) revealed a fibrous capsule with varying degrees of lymphocyte-rich inflammation adjacent to the LPS body and its tines (*Table 1*). The macroscopic encapsulation of the pacemaker was complete in two cases and partial (not covering the tail with the retrieval knob) in the other cases, and not associated with the length of the service period (*Figure 1*). The maximum thickness of the fibrous cast ranged between 465.1 µm and 1195.8 µm. CD34 immunohistochemistry showed that no endothelial cells covered the surface of the collagenous cast ‘membrane’. A brush lavage from the surface of the LPS in two patients (#6 and #7) confirmed this finding.

**Figure 1:**
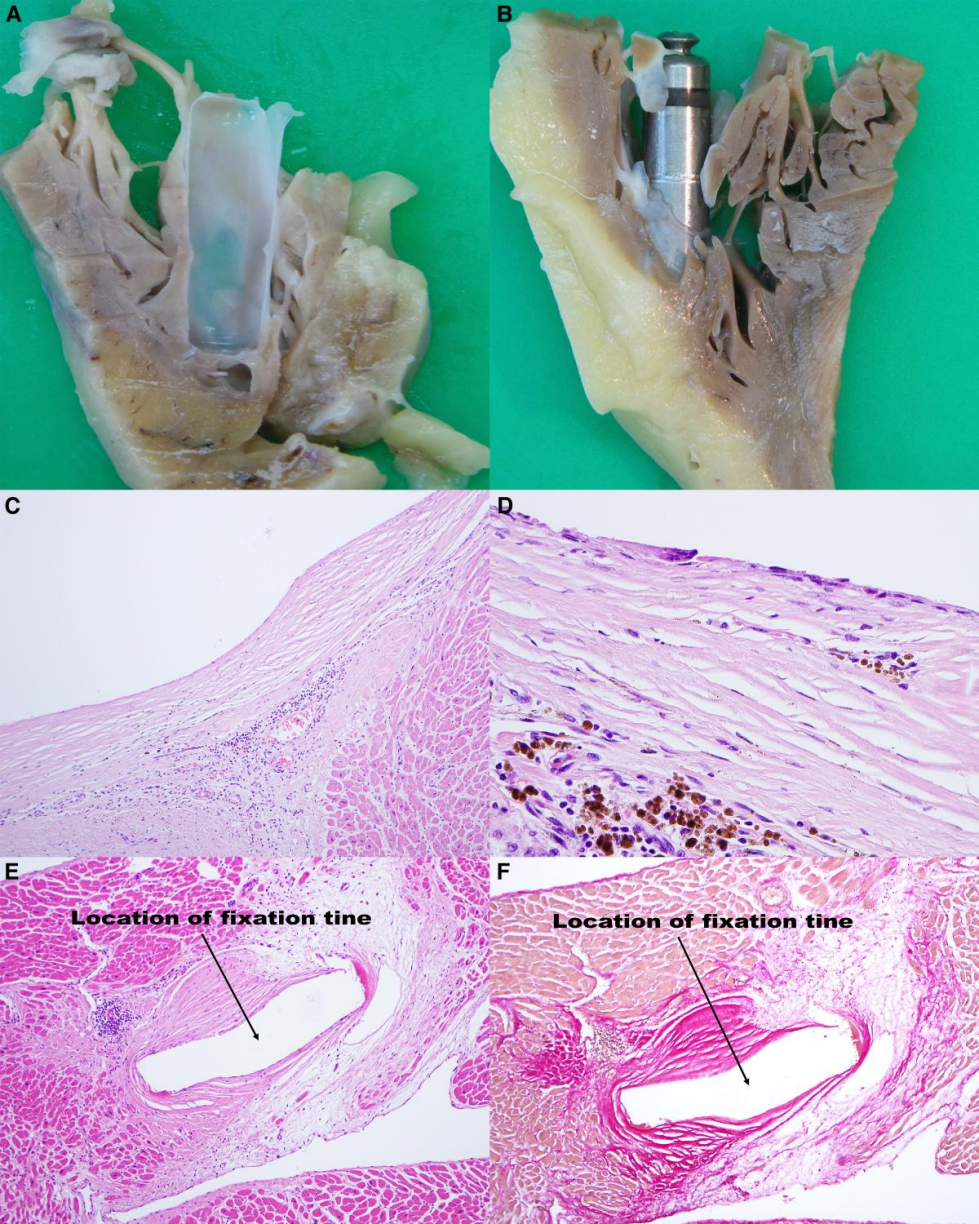
Macroscopic ingrowth patterns and microscopic views of the tissue surrounding the Micra™ LPS. (*A*) Cast totally covering the LPS (Patient #3, service period 697 days). (*B*) Cast only partially covering the LPS (Patient #1, service period 576 days). Stained sections of the tissue covering the device (LPS-tissue interface) with a deep layer of dense inflammatory cells (lymphocytes) covered by a capsule of collagenous tissue (cast, *C* and *D*). (*E* and *F*) Tissue reaction at the fixation tines. (*C*) Haematoxylin/eosin stain. Fibrous cast capsule with small blood vessels and lymphocytes. (*D*) Immunohistological stain for CD3 (T-lymphocytes). (*E*) Haematoxylin/eosin stain. Fibrous tissue reaction surrounding a fixation tine. (*F*) Elastica-Van-Gieson stain. Collagen red, myocardium yellow. LPS, leadless pacemaker system.

**Table 1:** Histological features of eight autopsy cases

Case number	#1	#2^3^	#3	#4	#5	#6	#7	#8
**Baseline characteristics and macroscopic features**								
Age at death (years)	76.7	83.3	75.7	75.6	86.1	86.3	87.4	83.9
Sex	Male	Male	Female	Male	Male	Female	Male	Male
Indication	Afib with slow conduction	Afib with slow conduction	Afib with slow conduction	SSS	Afib with slow conduction	AVB III	AVB III	AVB III
Length of service period in years (days)	1.6 (576)	1.0 (355)	1.9 (697)	1.1 (403)	0.6 (228)	5.7 (2083)	0.05 (18)	4.1 (1498)
Cause of death	Myocardial infarction	Renal disease, pulmonary fibrosis	Ovarian cancer	Liver cirrhosis	Renal disease	Ischaemic cardiomyopathy	Pulmonary disease, conventional device infection with sepsis	Prostate cancer
Macroscopic tissue coverage of LPS	In part	Total	Total	In part	In part	In part	In part	In part
Neointima	No	No	No	No	No	No	No	No
Location of LPS	Apical	Apical	Septal	Apical	Septal	Apical	Septal	Septal
**Histological features of the cast**								
Fibrosis	++	++	+/++	++	++	++	+	++
Inflammatory infiltrate	++	++/+++	+	++	++	+	−/+	+++
CD3 pos. lymphocytes	++	+++	+	++	++	+	+/−	+++
CD20 pos. lymphocytes	−/+	+	−/+	−/+	+	+	−/+	+
Neutrophils	+	−/+	−	−/+	−/+	−	−	−
CD34 staining	−	−	−	−	−	−	−	−
Fibrin exudates	+	++	−	+++	+++	++	++	−
Vascular proliferation	++	+++	+/−	+/++	+++	+	−/+	+
Haemosiderin	++	++	−	−/+	+++	−	−	−
Maximal thickness (µm)	974.3	916.1	485.8	931.2	922.1	1195.8	465.1	823.4
Miscellaneous	None	Focal myocardial scarring next to LPS	None	None	Amyloidosis, focal myocardial scarring next to LPS	Small calcifications	None	Focal myocardial scarring next to LPS

+, mild; ++, moderate; +++, marked; −, not present.

LPS, leadless pacemaker system; SSS, sick sinus syndrome.

Our indications for LPS therapy were in agreement with current guidelines.^4,5^ Compared with previously published data, the initial implantation success rate (99.6% in our cohort vs. 99.2%^6^), the 30-day complication rate (3.5% in our cohort vs. 2.9%^6^), and the long-term device-related complication rate (4.6% in our cohort vs. 4.6%^7^) were within the same range. Despite the significantly longer follow-up in our study (median 2.7, up to 7.1 years), all-cause mortality in our cohort (25.1%) was lower than in the Micra™ CED study (34%), which had a follow-up of only two years.^7^ This may be explained—at least in part—by a lower burden of comorbidities in our set of patients, as reflected by a lower Charlson index (4.5 ± 1.6 points in our cohort vs. 5.1 ± 3.4^7^). However, the proportion of causes of death in our cohort (cardiac: 45.1%, non-cardiac: 54.9%) was quite comparable to the 12-month results of the Micra™ Transcatheter Pacing Study (cardiac: 41.6%, non-cardiac: 55.8%, unknown: 2.6%).^6^ In both our cohort and in the Micra™ LPS 12-month follow-up study, one death occurred during the index stay that was not related to the LPS implantation procedure (due to sepsis in our cohort).

The fibrotic reaction seen in our histopathological analyses is most likely due to direct implantation trauma and mechanical irritation of the right ventricular wall and trabeculae by the LPS.^8,9^ It has been shown *in vitro* that IL-1β and IL-6, which act on fibroblasts and endothelial cells, are important for mediating this tissue reaction.^10^ In our cohort, maximum cast thickness and extent of the macroscopic overgrowth were unpredictable and not associated with the length of the service period. Conversely, they probably depend more on the individual predisposition to scar formation, which is underlined by our observation that the LPS is either already fully encapsulated one year after implantation^3^ or is not yet completely covered by tissue after 5.7 years. In a porcine model, after 12 weeks, the extent of fibrosis was significantly less with the Micra™ LPS than with conventional Medtronic™ 5076 leads.^11^ Micra™ retrievals described in the literature were feasible up to four years after implantation but were technically demanding in some cases.^12–15^ We could not identify endothelial lining with CD34 staining at the device-tissue interface. Such an ‘acellular interface’ was also found in an analysis of collagen-rich connective tissue surrounding standard pacemaker leads.^8^ In addition to the fibrotic cast, we found T-cell-rich inflammation with fibrin exudates, haemosiderin, and vascular proliferation adjacent to the LPS, indicating wound healing, scarring, and an individual reaction to foreign material.

## Data Availability

The data presented in this study will be shared upon reasonable request to the corresponding author.
